# Selecting a single model or combining multiple models for microarray-based classifier development? – A comparative analysis based on large and diverse datasets generated from the MAQC-II project

**DOI:** 10.1186/1471-2105-12-S10-S3

**Published:** 2011-10-18

**Authors:** Minjun Chen, Leming Shi, Reagan Kelly, Roger Perkins, Hong Fang, Weida Tong

**Affiliations:** 1 Center for Bioinformatics, Division of Systems Biology, National Center for Toxicological Research, U.S. Food & Drug Administration, 3900 NCTR Rd, Jefferson, Arkansas, USA; 2 ICF International at FDA's National Center for Toxicological Research, 3900 NCTR Rd, Jefferson, AR 72079, USA

## Abstract

**Abstract:**

**Background**

Genomic biomarkers play an increasing role in both preclinical and clinical application. Development of genomic biomarkers with microarrays is an area of intensive investigation. However, despite sustained and continuing effort, developing microarray-based predictive models (i.e., genomics biomarkers) capable of reliable prediction for an observed or measured outcome (i.e., endpoint) of unknown samples in preclinical and clinical practice remains a considerable challenge. No straightforward guidelines exist for selecting a single model that will perform best when presented with unknown samples. In the second phase of the MicroArray Quality Control (MAQC-II) project, 36 analysis teams produced a large number of models for 13 preclinical and clinical endpoints. Before external validation was performed, each team nominated one model per endpoint (referred to here as 'nominated models') from which MAQC-II experts selected 13 'candidate models' to represent the best model for each endpoint. Both the nominated and candidate models from MAQC-II provide benchmarks to assess other methodologies for developing microarray-based predictive models.

**Methods**

We developed a simple ensemble method by taking a number of the top performing models from cross-validation and developing an ensemble model for each of the MAQC-II endpoints. We compared the ensemble models with both nominated and candidate models from MAQC-II using blinded external validation.

**Results**

For 10 of the 13 MAQC-II endpoints originally analyzed by the MAQC-II data analysis team from the National Center for Toxicological Research (NCTR), the ensemble models achieved equal or better predictive performance than the NCTR nominated models. Additionally, the ensemble models had performance comparable to the MAQC-II candidate models. Most ensemble models also had better performance than the nominated models generated by five other MAQC-II data analysis teams that analyzed all 13 endpoints.

**Conclusions**

Our findings suggest that an ensemble method can often attain a higher average predictive performance in an external validation set than a corresponding “optimized” model method. Using an ensemble method to determine a final model is a potentially important supplement to the good modeling practices recommended by the MAQC-II project for developing microarray-based genomic biomarkers.

## Background

Gene expression microarrays have been applied in various fields [1-6]. Despite widespread usage, the translation of basic findings to clinical utility such as diagnosis and prognosis has been slow. This is largely due to the fact that some clinical endpoints are difficult to predict with microarrays, such as prediction of drug-induced liver injury [7], and survival endpoints for many cancers [8]. In addition, issues such as small sample size, low signal-to-noise ratio and lack of a fully annotated transcriptome contribute to the lack of success in developing biomarkers (i.e., predictive models or classifiers) with microarrays [9,10].

The conventional procedure of developing a microarray-based biomarker involves a selection process to identify one classifier out of many others generated in this process for application to an external dataset. The selection is largely dependent on the accuracy estimation [11]. Specifically, the “optimized” model is selected using the training set with, for example, cross-validation to estimate its predictive performance. Some authors argue that cross-validation can provide an unbiased estimate of performance when properly applied [12,13] while others point out that the variability in the error estimation can be very high when cross-validation is applied to datasets with small sample sizes [14]. Thus, there exists a great uncertainty that an accuracy-based model selection procedure will choose the best microarray-based classifier [12,15].

Selecting a single optimized model is the most common approach to developing microarray-based predictive models [6,16-18]. However, it is being challenged given the fact that many models with similar statistical performance are often identified for a studied endpoint. By reanalyzing the breast cancer prognosis dataset reported by van’t Veer *et al.*[8], Ein-Dor *et al.* noticed that many gene sets gave nearly equal prediction accuracy [19]. The question is whether the combination of these well performing models could be preferable to an accuracy-based selection of a single optimized model from among many.

Ensemble methods have been demonstrated its usage in some fields such as machine learning [20] and Quantitative Structure Activity Relationships (QSAR) [21]. These investigations are carried out under the hypothesis that the methods likely capture a greater diversity of potentially informative features [21] that might improve the model robustness when included. Ensemble methods have similarly been explored in gene expression studies [22,23]. It was found that enhanced prediction accuracy for ensemble methods compared to the single model selection method, especially for complex and/or heterogeneous endpoints. However, the comparative analysis was carried out on limited datasets sometimes having small sample sizes. A rigorous comparison where findings can be generalized is best achieved with a systematic comparative analysis using multiple datasets containing endpoints with different characteristics. The second phase of MicroArray Quality Control (MAQC-II) project, led by the U.S. Food and Drug Administration with broad participation from the large research community, offers the benchmark data to allow such a rigorous comparison.

One goal of the MAQC-II project was to develop baseline practices for the application of microarray technology to biomarker development [24]. This process took nearly four years to enable a full investigation of the impact of modeling procedure choices on the quality of predictive models. The project provides the requisite datasets as well as a large number of validated models developed using diverse methods for comparison. Specifically, the 36 analysis teams generated more than 30,000 models across 13 endpoints from six datasets. Importantly, similar prediction performance was attained despite the use of different modeling algorithms and gene sets. However, the MAQC-II required each team to first nominate and then validate in blinded manner a single model (or nominated model) for each endpoint. A group of experts then selected 13 final models (one per endpoint) that were designated candidate models. The performance of these selected ‘optimized’ models (both nominate and candidate models) was assessed on blinded, independent validation sets. The comprehensive and disciplined process employed by this approach in selecting optimized models resulted in a set of nominate and candidate models constituting sound benchmarks for comparison of ensemble methods.

In this study we applied a simple ensemble approach of combining the top 50% of all the models from the selected MAQC-II team and compared them with the nominated and candidate models for each endpoint (More details can be found in Results.). In other words, we took the simplest way to generate ensemble models and then compare them with the optimized models generated from the most sophisticated and comprehensive approaches implemented in MAQC-II. Our study indicates that even such simple ensemble methods can achieve comparable if not better predictive performance in external validation sets than the corresponding single “optimized” model from MAQC-II.

## Methods

### Datasets

All six MAQC-II datasets were used for this study [24]. Three datasets are related to toxicogenomics endpoints: lung tumorigenicity from Thomas, *et al.*[25], non-genotoxicity in the liver from Fielden *et al.*[26], and liver toxicity from the Lobenhofer *et al.*[27]. The remaining three datasets are related to cancer prognosis for breast cancer [28], multiple myeloma [29,30], and neuroblastoma [31]. Together they contain 13 preclinical and clinical endpoints, designated A through M, as shown in Table 1. Endpoints I and M are “disguised” negative controls with randomly assigned classes, and endpoints H and L are “disguised” positive controls representing patient sex; “disguised” indicates here that the MAQC-II analysis teams did not know the nature of these endpoints. There are three preclinical endpoints assessing toxicity (A, B and C), and six clinical endpoints (D, E, F, G, J and K) representing patient responses in breast cancer, multiple myeloma or neuroblastoma. The training sets were provided to the analysis teams to develop models using the known labels. Once all the analysis teams had developed the final models and frozen them, the validation sets were released with blinded labels. Details about the datasets, the experimental design and timeline of the MAQC-II project are discussed by Shi, *et al*.[24].

**Table 1:** The datasets used in MAQC-II project.

Endpoint code	Endpoint	Endpoint description	Training set		Validation set	
	
			#Sample	P/N ratio*	#Sample	P/N ratio
A	Lung tumorigenicity	Lung tumorigen vs. non-tumorigen	70	0.59	88	0.47
B	Non-genotoxicity	Non-genotoxic hepatocarcinogen vs. non-carcinogen	216	0.51	201	0.4
C	Liver toxicity	Liver toxicants vs. non-toxicants	214	0.58	204	0.62
D	Breast cancer	Pathologic complete response, pCR	130	0.34	100	0.18
E	Breast cancer	Estrogen receptor status (ER +/-)	130	1.6	100	1.56
F	Multiple myeloma	Overall survival	340	0.18	214	0.14
G	Multiple myeloma	Event-free survival	340	0.33	214	0.19
H	Multiple myeloma	Male vs. female (positive control)	340	1.33	214	1.89
I	Multiple myeloma	Random 2-class label (negative control)	340	1.43	214	1.33
J	Neuroblastoma	Overall survival	238	0.1	177	0.28
K	Neuroblastoma	Event-free survival	239	0.26	193	0.75
L	Neuroblastoma	Male vs. female (positive control)	246	1.44	231	1.36
M	Neuroblastoma	Random 2-class label (negative control)	246	1.44	253	1.36

* P/N = Positive/Negative ratio. Positive denotes for these samples showing the positive results (e.g. cancer, tumor).

### Data analysis protocol of NCTR models

**Figure 1:**
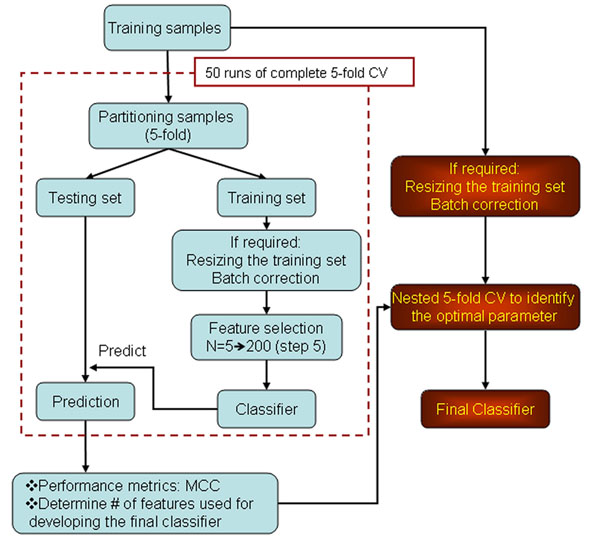
Overview of the NCTR model development process.

The analysis team from the National Center for Toxicological Research (NCTR) was one of the 36 analysis teams in the MAQC-II consortium. The models used in this study were those generated during the MAQC-II process with no retroactive modification. This section describes the original analysis that was done as depicted in Figure 1 flowchart. The center mean shift approach was applied to correct potential batch effect. For datasets having a skewed class ratio greater than four, the class distribution was balanced by over sampling the minority class to attain an even distribution. The two statistical methods, fold change plus p-value (from a simple t-test) and Significance Analysis of Microarrays (SAM) [32], were used for feature selection beginning with the training datasets. In the fold change plus p-value method, the features were chosen by first ranking genes by absolute fold change, and then excluding all features that did not satisfy p-value <0.05. The top five features were included first in the cross-validation (CV) and the process was then repeated by incrementally adding five features more at each step until the number of features reached 200. In the SAM method, a relative difference defined in the SAM algorithm was applied to rank the features, followed by a feature selection approach analogous to fold-change plus p-value. We applied two different classification methods, k-nearest neighbors (KNN) and Naive Bayes modeling, to develop models for each of the 13 endpoints. For a range of parameter values, 8320 models were developed and submitted to the MAQC-II consortium, including 7280 KNN models (i.e., 13 endpoints × 40 features sets × 2 feature selection methods × 7 parameters of K) and 1040 Naïve Bayes models (i.e., 13 endpoints × 40 features sets × 2 feature selection methods). The classification methods were applied using R [33] and the klaR package [34].

### Selection of NCTR nominated models

A complete 5-fold CV procedure was employed to determine the number of features and modeling parameters used to develop the final classifier. The complete CV embeds the entire modeling process including, batch correction, resizing training set and feature selection in each of the cross-validation steps. The average performance of the classifiers from the 50 CV runs was calculated and the parameters that resulted in the best classifier were used for developing the final classifier using the entire training set. As recommended by the MAQC-II consortium [24], MCC (Mathhews Correlation Coefficient) was the selected metric for assessing model performance. An MCC-guided method was used to identify the models to be submitted for each endpoint, which consisted of a hierarchical decision tree with a knowledge justification at each level of the decision. Specifically, the following step was used:

• Step 1 – Decision based on the MCC value: The MCC value was adjusted to one decimal precision and models with the same MCC value were grouped. For example, models with MCC values of 0.89 and 0.91 were considered as performing equally and placed into the MCC > 0.9 group. The models in the group with the highest MCC value were passed to the next step.

• Step 2 – Decision based on the number of features: Within a group of models with the same MCC value, more parsimonious models were given higher priority. However, if two models contained nearly the same number of features, then accuracy, sensitivity and specificity were used to choose the best performing model.

• Step 3 – Decision based on the feature selection method: For equally well-performing models, those that used SAM for feature selection were chosen over those that used fold change plus p-value.

• Step 4 – Decision based on the classification method: For equally well-performing models those created using KNN were selected over those created using a Naive Bayes classifier.

### Ensemble method

An ensemble model was developed for each endpoint for comparison to those submitted for MAQC-II evaluation. An ensemble model was derived by taking the 50% of the models from cross-validation with the highest MCC and using a voting process to make a final prediction about a sample. To begin, the average percentage of positive predictions for the 50% of models in the training set is recorded. For each sample in the validation set, the percentage of models producing a positive prediction is calculated. This percentage is then divided by the average percentage of positive predictions in the training set recorded earlier. If the ratio of these numbers is one or greater, the ensemble model will produce a positive prediction. Otherwise the ensemble model will give a negative prediction. External validation was done while blinded to the class of the external test sets as implemented in MAQC-II.

## Results

	As one of the 36 analysis teams involved in the MAQC-II project, we generated 8320 models (7280 KNN models and 1040 Naïve Bayes models). As shown in Additional file 1, the correlation coefficient (r=0.927) of our submitted models in the external validation was higher than that from all the MAQC-II models (r=0.840) [24], indicating that the performance of our models was above the average among the 36 analysis teams. We also selected one model per endpoint (called the NCTR nominated models) from all the NCTR models using the accuracy-based selection method (refer to the Methods sections for more details). Meanwhile, each analysis team that participated in the project also nominated one model for each endpoint they analyzed according to the MAQC-II guidance. An MAQC-II expert committee then selected 13 candidate models, representing the best model for each endpoint from all the submitted models from the 36 teams, before external validation was performed. In this study, the ensemble models comprising of 50% of all the NCTR models with highest performance in cross-validation are against both the NCTR nominated models as well as the MAQC-II candidate models across all the 13 endpoints. To validate the findings from the NCTR-centric practice, the same analysis was carried out on the models generated by other analysis teams. Comparative assessment was based on the blinded external validation performance.

### The NCTR ensemble models vs. the NCTR nominated models

**Figure 2:**
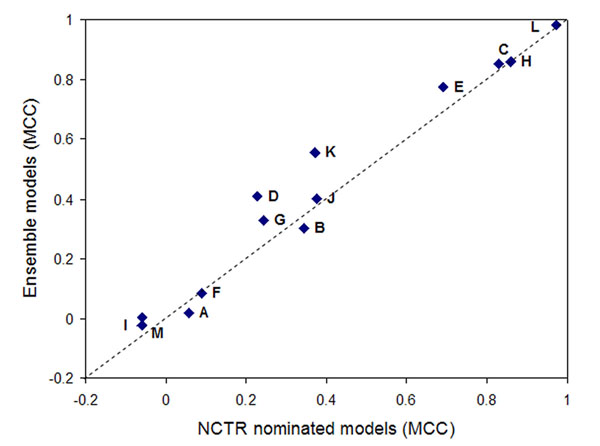
**The ensemble models vs. the NCTR nominated models.** A pair-wise t-test was applied to the MCCs obtained from the ensemble models and the NCTR nominated models. (P-value = 0.039 if two random endpoints, i.e., I and M, were excluded).

As shown in Figure 2, using MCC as the performance metrics we found that for 10 of the 13 endpoints, the ensemble model achieved a better or equal MCC value than the NCTR nominated model. The pair-wise t-test indicated that the average MCC of the ensemble models was significantly higher than the average from the NCTR-nominated models (P-value = 0.039 if two random endpoints, i.e., I and M, were excluded).

**Figure 3:**
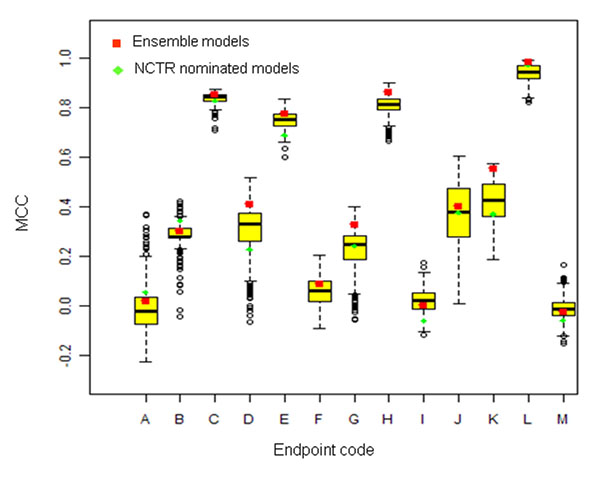
**The ensemble models and the NCTR nominated models related to all the NCTR developed models.** The distribution of the cross-validation MCCs from 8320 NCTR developed models for each endpoint was shown in the box plots; the NCTR nominated models were marked as the green diamonds, and the ensemble models were marked as the red squares.

We also compared the MCC of the NCTR nominated models and the NCTR ensemble models in the external validation sets to that of the full set of developed models (N=8320). The box plots in Figure 3 show the MCC distribution for these models for each of the 13 endpoints, with the NCTR nominated model shown as a green diamond and the ensemble model shown as a red square. For a well-performing model selection method either the "optimized" or the ensemble model or both should be better than the median MCC in the external validation set for the full set of developed models for a particular endpoint. For the endpoints C, D, E, G, J, and K, the NCTR nominated models showed an MCC below the median value of all developed models. In contrast, none of the ensemble models had an MCC below the median value. Moreover, the MCCs of the ensemble models for endpoints of C, D, E, F, G, H, K, and L ranked in the top 25% of values from all selected models, and those for endpoints of A, B, and J also ranked above the median value of all developed models. This is a strong evidence that the ensemble models provides more consistent performance in the external validation set than the NCTR nominated models selected based on the accuracy.

### The NCTR ensemble models vs. the MAQC-II candidate models

**Figure 4:**
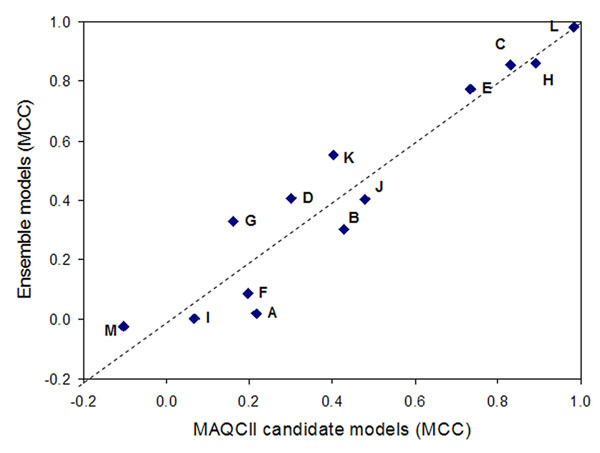
**The ensemble models vs. MAQC-II candidate models.** A pair-wise t-test was applied to the MCCs obtained from the ensemble models and the MAQC-II candidate models (P-value = 0.43).

We compared the NCTR ensemble models with the MAQC-II candidate models. The candidate model for each endpoint was selected by the MAQC-II expert group from among the nominated models submitted by the 36 data analysis teams before the external validation, which represented the best practice to select an “optimized” model. As shown in Figure 4, there is no significant difference in the average MCC values between the NCTR ensemble models and the candidate models (P-value = 0.43 from a pair-wise t-test), indicating that the simple ensemble method used in this study can achieve equivalent performance of those selected by the experts.

### The comparative analysis of models from different MAQC-II analysis teams

**Figure 5:**
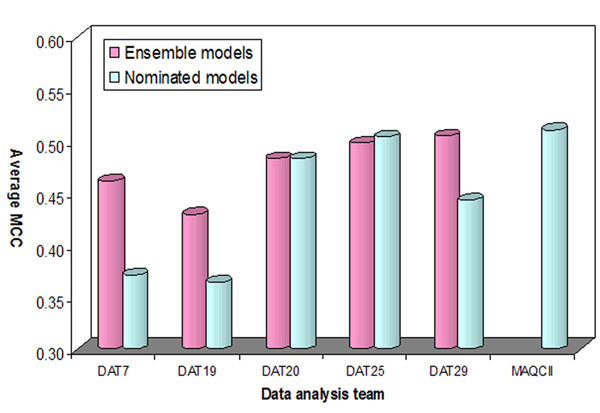
**The comparison of the models from different analysis teams.** The average MCC was calculated from 11 non-random endpoints in the external validation sets when I and M were excluded.

We performed the same comparative analysis for the models generated by other analysis teams. We selected only those teams that analyzed all 13 endpoints and submitted more than 260 models. This resulted in only 5 teams (DAT7, DAT19, DAT20, DAT25, and DAT29 as denoted by the consortium). We generated the ensemble models for each endpoint modeled by each team and the results were compared with their nominated models as well as the candidate models. The average MCC from the 11 non-random endpoints (i.e., excluding endpoints I and M) was used for the comparison. As depicted in Figure 5, most ensemble models performed better than the corresponding nominated models. The exceptions were DAT20 and DAT25 as they demonstrated similar performance of the ensemble and nominated models.

## Discussion

Microarray-based models to predict preclinical and clinical endpoints have become routine in research. Most studies focus on the selection of a single “optimized” model, but it is never clear whether that model will provide acceptable performance on an external validation set. Given the fact that many models from the same training set could achieve similar predictive performance, we investigated a simple ensemble approach of combining the top 50% best performing models and compared it with the single model selection approach. We conducted the investigation using the MAQC-II results because the MAQC-II project (1) covers a diverse set of endpoints including both “disguised” positive and negative controls, offering an opportunity to examine the issue in a systematic fashion; (2) generated the results from the blinded validation sets with large sample sizes, an important criterion to ensure the validity of the investigation; (3) provides the nominated models from each of 36 analysis teams, which represents a broad range of model selection methods; and (4) yielded the MAQC-II candidate models, representing the “best practice” of developing classifiers using the model selection method.

Using the MAQC-II results from the NCTR team and validated by the results from other five MAQC-II data analysis teams, two important observations were made. First, within each team, the ensemble method consistently generated models performing better in the external datasets than the model selection methods implemented by different teams. Second, the ensemble method performed comparably to the MAQC-II candidate models that were chosen with considerable efforts. The results demonstrate that identification of a single best model solely based on the statistical performance is difficult as exemplified in the MAQC-II nominated models where knowledge and experience behind the model selection is crucial as practiced in the determination of the MAQC-II candidate models. The proposed ensemble approach is easy, objective and reproducible, and thus can be an alternative method to generate a robust model based on the training set.

Accuracy estimation of a classifier using only a training set is still a difficult issue due to over-fitting, which is one of the major limitations associated with predictive models. Models often have excellent performance in the training dataset but nonetheless poorly predict in external validation datasets, even when best modeling practices are employed. The inconsistent predictive performance between the training set and testing set stems from the influence of idiosyncratic associations between features and endpoints in the training set. Cross-validation is a common method to account for these idiosyncrasies and to estimate accurately the prediction error of the models. Simon *et al*. proposed that the cross-validation, if used properly [13], provides a nearly unbiased estimate of the true error of classification procedure, while incomplete cross-validation will result in a seriously biased underestimate of the error rate. From our experience in the MAQC-II consortium, we found that the accuracy based selection process, even using the complete cross-validation procedure, still lead to models that are apparently over-fit and perform poorly on the external datasets (Additional file 2). In other words, a degree of over-fitting still exists even after properly applying "complete" cross-validation. This demonstrates that reducing the risk of over-fitting is still an issue in the selection method that must be addressed in order to improve the performance of microarray-based predictive models.

In this study, during cross-validation it was observed that many models could attain similar performance, while the models that produced the best MCCs in the training sets did not necessarily provide the best MCCs in the external validation sets. Based on these observations, it is reasonable to assume that an ensemble modeling method could substantially mitigate the risk of over-fitting presented in the “optimized” model selection process, although the ensemble models could not always generate *the* best predictive model.

Ensemble models have been well studied in the machine learning area where they have been shown useful for improving prediction performance [35]. Random forest is a representative algorithm that consists of many decision trees that vote to select class membership. Some authors also reported that ensemble methods have worked well in QSAR models [21,36] and microarray-based studies [22,23] with a small number of datasets, but a literature search did not produce any comprehensive evaluations of the utility of ensemble methods in microarray-based classifier development. The MAQC-II study participants did not determine a preferred approach to select a best model for each endpoint, leaving that selection as part of an individual team’s preference. The data reported here did support this conclusion; in 8 of the 11 non-random endpoints (i.e., excluding endpoints I and M) the ensemble models were ranked in the top 25% of MCC values from all of the developed models.

It should also be noted that the choice to use the top 50% models based on cross-validation for the ensemble models was arbitrary. The data from further experiments shown in Additional file 3 have suggested that the choice of the number of models to be combined does not greatly affect performance of an ensemble model as long as a sufficient number of models (e.g., > 10% models) are retained in the process. The combination of too many models will actually decease slightly the performance, likely because of the noise introduced by the models with relatively poor performance. In contrary, using too few models does not have too much value due to the lack of representative models in ensemble. Therefore, we suggest that a modest number of models should be retained for ensemble calculation.

Many factors affect the performance of the microarray-based classifiers. The MAQC-II consortium comprehensively evaluated most of these factors through a community-wide practice, and established good modeling practice guidelines [24]. This study provides a follow-up and extension of the MAQC-II team efforts. We found that an ensemble modeling procedure can reduce the risk of over-fitting and provides stable and robust predictive power than those single “optimized” models. These findings provide a necessary supplement to the good modeling practices for developing microarray-based predictive classifiers developed in the MAQC-II process.

## List of abbreviations used

MAQC: MicroArray Quality Control; NCTR: National Center for Toxicological Research; QSAR: Quantitative Structure Activity Relationship; SAM: Significance Analysis of Microarrays; CV: Cross-validation; KNN: K-nearest neighbors; MCC: Matthews Correlation Coefficient.

## Competing interests

The authors declare that they have no competing interests.

## Authors' contributions

MC performed all calculations, data analysis, and wrote the first draft of manuscript. LS and WT developed the methods, conceived the original idea, and guided the data analysis. RK, RP, WT and HF contributed to the data analysis, verified the calculations, and assisted with writing the manuscript. All authors read and approved the final manuscript.

## Disclaimer

The views presented in this article do not necessarily reflect those of the US Food and Drug Administration.

## Supplementary Material

Additional file 1 **Internal cross-validation vs. external validation of the 8320 NCTR developed models.** The Pearson correlation of MCCs from Internal cross-validation vs. external validation is 0.927. Click here for file

Additional file 2 Internal cross-validation vs. external validation of the NCTR nominated models. Click here for file

Additional file 3 **The average MCCs vs. the percentages of the top models for ensemble calculation.** The average MCC was calculated from 13 endpoints in the external validation set; the top models were selected based on the MCCs from internal cross-validation in the training set. Click here for file
