# Supplementary figures and images for: Selecting a single model or combining multiple models for microarray-based classifier development? – A comparative analysis based on large and diverse datasets generated from the MAQC-II project
Source: BMC Bioinformatics. 2011 Oct 18;12(Suppl 10):S3. doi: 10.1186/1471-2105-12-S10-S3 (PMC3236846; doi:10.1186/1471-2105-12-S10-S3)

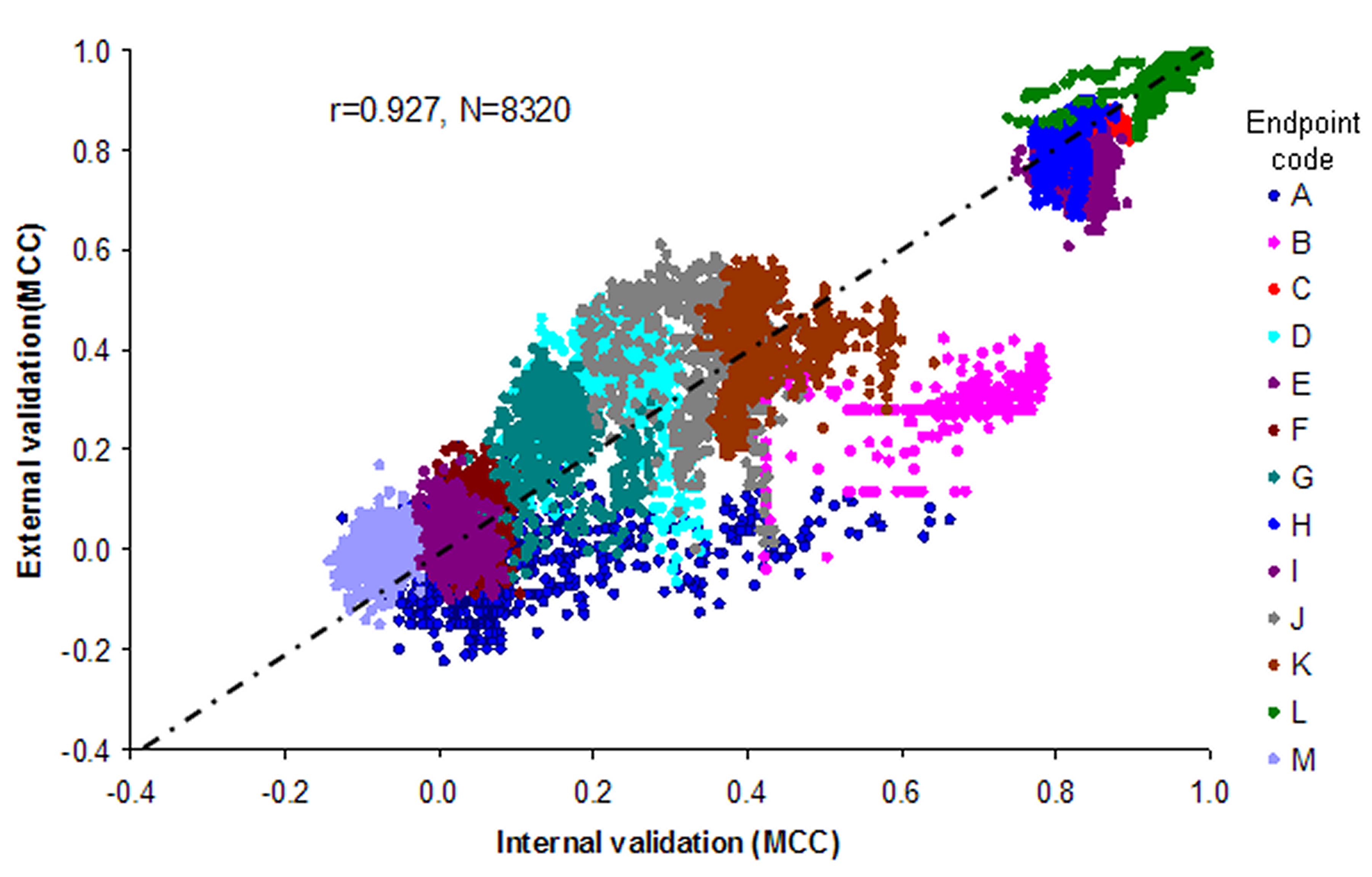

Supplement: Additional file 1 — Internal cross-validation vs. external validation of the 8320 NCTR developed models. The Pearson correlation of MCCs from Internal cross-validation vs. external validation is 0.927. [file 1471-2105-12-S10-S3-S1.bmp]

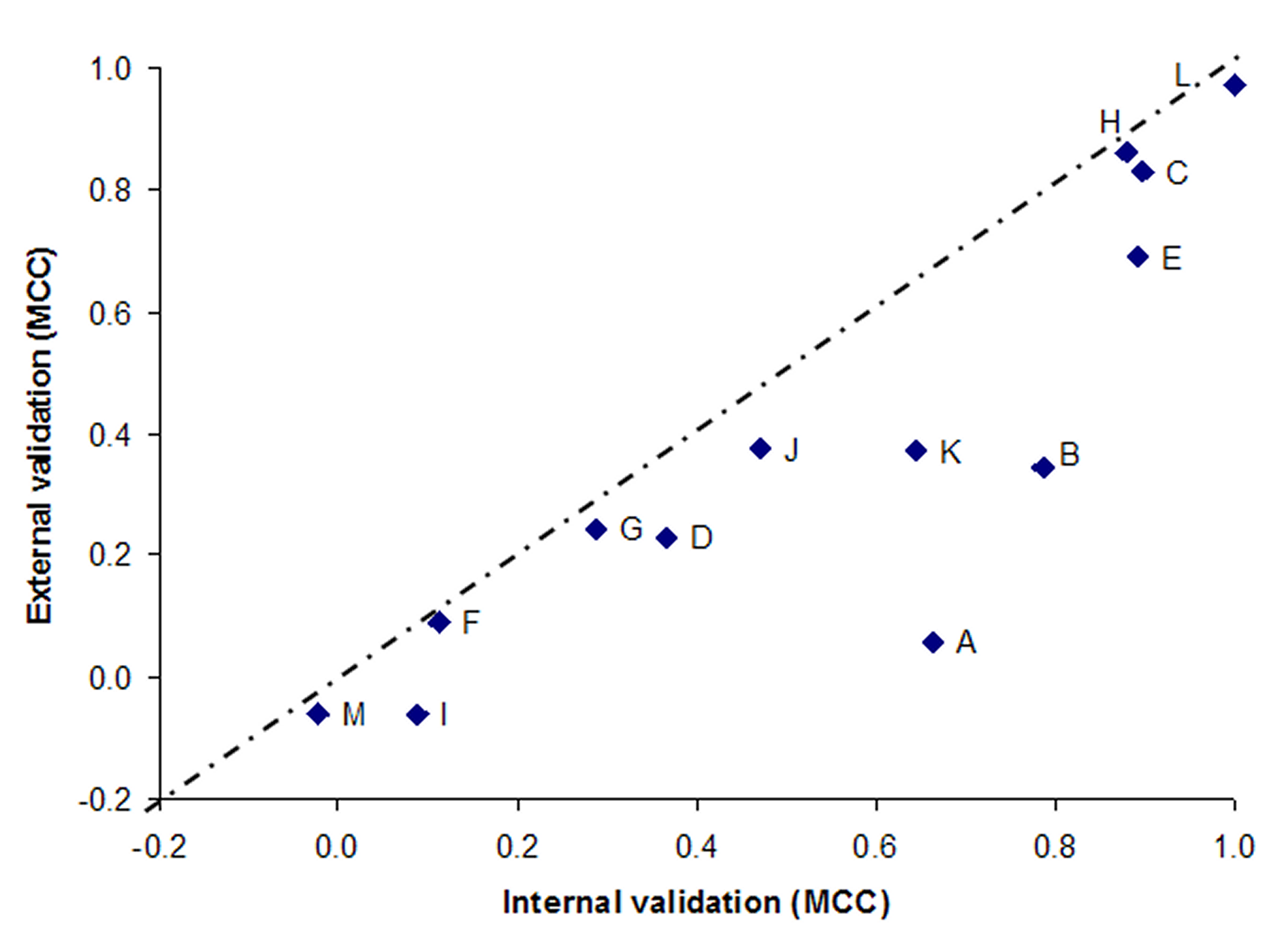

Supplement: Additional file 2 — Internal cross-validation vs. external validation of the NCTR nominated models. [file 1471-2105-12-S10-S3-S2.bmp]

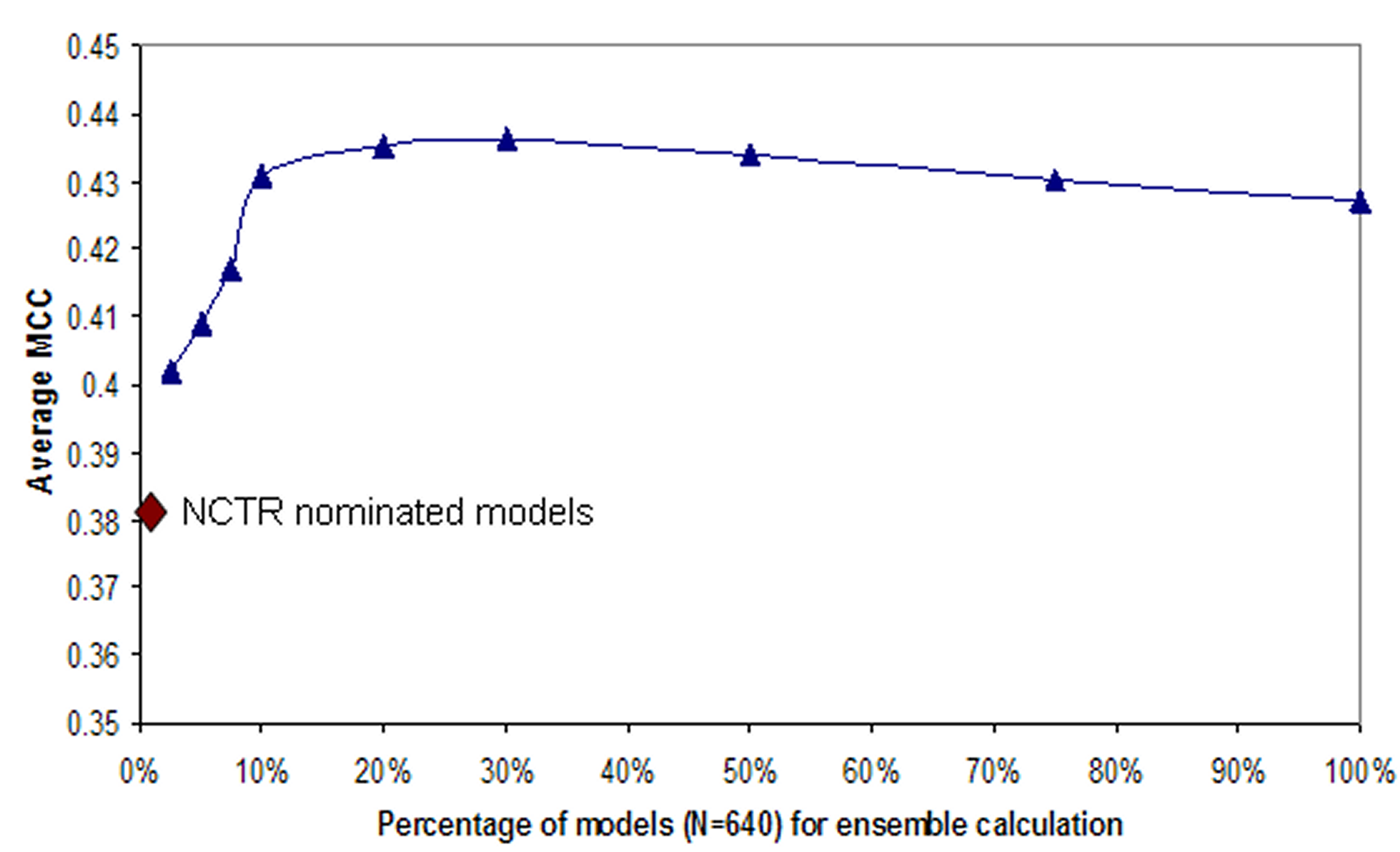

Supplement: Additional file 3 — The average MCCs vs. the percentages of the top models for ensemble calculation. The average MCC was calculated from 13 endpoints in the external validation set; the top models were selected based on the MCCs from internal cross-validation in the training set. [file 1471-2105-12-S10-S3-S3.bmp]
